# Supplementary material for: Traditional Beliefs, Practices, and Migration: A Risk to Malaria Transmission in Rural Nepal
Source: Int J Environ Res Public Health. 2022 Dec 15;19(24):16872. doi: 10.3390/ijerph192416872 (PMC9779137; doi:10.3390/ijerph192416872)
Supplement: Supplementary file 1 [file ijerph-19-16872-s001.zip › ijerph-2027653-supplementary.pdf]

Supplementary File: Traditional beliefs, practices and migration: A risk to malaria transmission in rural Nepal

Kiran Raj Awasthi [1,*], Jonine Jancey [1], Archie C. A. Clements [1,2,3], Rohit Kumar Sah [4], Madan Prasad Koirala[4], Binaya Chalise[5], Justine E. Leavy [1]

1 Curtin School of Population Health, Curtin University, GPO Box U1987, Perth, WA 6845, Australia
2 Telethon Kids Institute, Perth, WA 6009, Australia
3 Peninsula Medical School, University of Plymouth, Plymouth PL4 8AA, UK
4 National Malaria Program, Karnali Province Field Office, Nepalgunj, 21900, Nepal
5 Graduate School for International Development and Cooperation, Hiroshima University, Higashi-Hiroshima 739-8529, Japan
* Correspondence: kiran.awasthi@curtin.edu.au

Figure S1: Qualitative Themes Overview

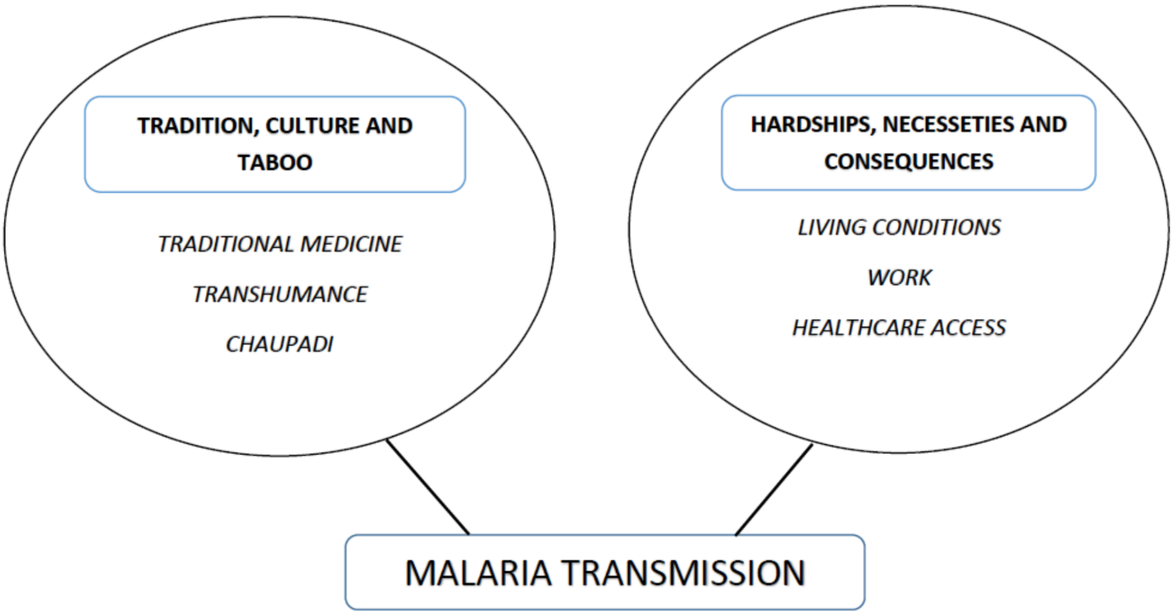

Figure S2: Chhaupadi and Chaugoth

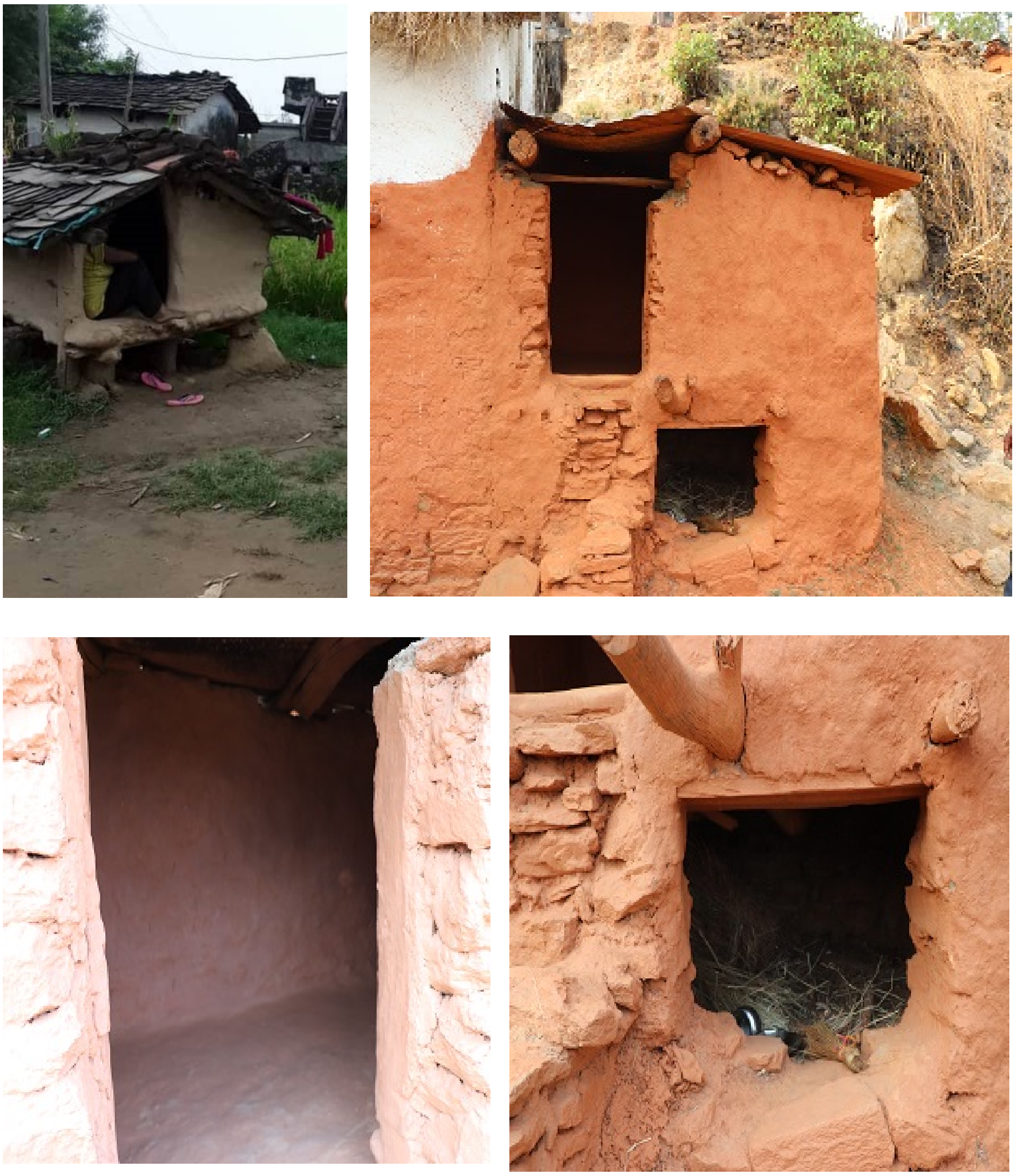

Source: KRA, Photos were taken on 16th November (top left) 2018 and 28th October 2021 (remaining three)

Description: The first photo (tope left) shows a woman staying in a Chaugoth during her menstruation. The remaining three pictures show a typical Chuagoth in Mugu. As seen in the second picture (top right), there is no door or proper ventilation. The third picture (below left) shows the minimal space to allow the use of LLINs or ITNs. The fourth picture (below right) shows the proximity of the goat shed to the Chaugoth.

Figure S3: Traditional dwellings in Rigga village, Nepal

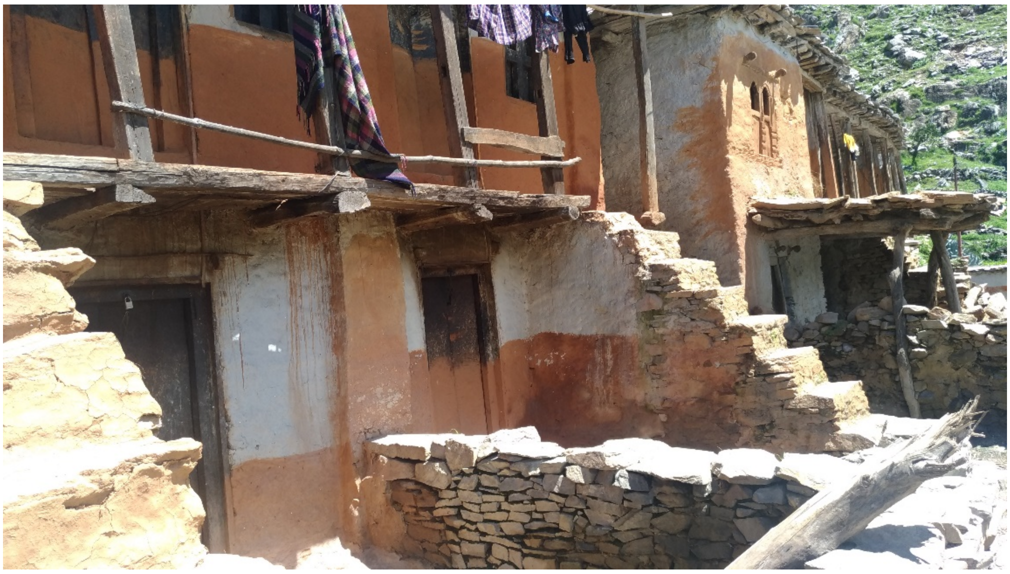

Source: KRA, Photo was taken on 25th April 2018
Description: The above photo shows a typical village house in Rigga village, the study site. The bottom floor is the cow shed whilst people live on the first floor.

Table S1: One-on one interview participants characteristics (n=25)

Demographics	(n)
**Sex**	
Male	**10**
Female	**15**
**Type of family**	
Nuclear family (less than two generations in one house)	**10**
Extended family (three or more generations in one house)	**15**
**Family size**	
Small family (< 5 members)	**5**
Large Family (≥ 5 members)	**20**

Table S2: Household survey: socio-demographics, beliefs towards malaria treatment, attitude, and sleeping practices (n=218)

Demographics	n (%)
**Sex**	
Male	108 (49.5)
Female	110 (50.5)
**Age (years)**	
15-30	55 (25.2)
31-40	48 (22.0)
41-50	34 (15.6)
>50	81 (37.2)
**Occupation**	
Business	3 (1.4)
Daily wage worker/ migrant worker	50 (22.9)
Agriculture	120 (55)
Housewife	28 (12.8)
Salaried job (govt/private)	12 (5.5)
Student	5 (2.3)
Inside	217 (99.5)
Outside	1 (0.05)
**Education**	
Primary school (Grade 5)	24 (11.0)
Secondary school (Grade 10)	29 (13.3)
Higher Secondary School (Grade 12)	19 (8.7)
Literate (able to read and write only)	146 (67.0)
**Monthly family Income (1 USD= 132 NRs)**	
<10,000 NRs	185 (84.9)
10,000-25000 NRs	27 (12.4)
25,000-50,000 NRs	4 (1.8)
>50,000 NRs	2 (0.9%)
**Type of family**	
Nuclear family (less than two generations in the house)	117 (53.7)
Joint family (three or more generations in the house)	101 (46.3)
**Family Size**	

Small family (<5 members)	104 (47.7)
Large family (6-10 members)	98 (45.0)
Very large family (>11 members)	16 (7.3)
**Key decision makers in the family**	
Father-in-law	19 (18.7)
Mother-in-law	8 (3.7)
Husband	132 (60.6)
Wife	6 (2.8)
Others	9 (4.1)
**Beliefs towards malaria treatment**	
**Malaria can be cured?**	
No	4 (1.80)
Yes	214 (98.2)
**The deciding factor when seeking care for malaria?**	
Condition of the patient	110 (50.5)
Condition of the patient, cost involved	23 (10.5)
Condition of the patient, cost involved, availability of time	1 (0.50)
Cost involved	84 (38.5)
**I can treat myself if I get malaria**	
Agree/strongly agree	33 (15.1)
Disagree/strongly disagree	185 (84.9)
**It is dangerous if malaria medication is not completed as prescribed**	
Agree/strongly agree	202 (92.7)
Disagree	14 (6.4)
Neither agree nor disagree	2 (0.9)
**Attitude**	
**I can buy antimalarial drugs from the pharmacy to treat myself.**	
Agree/ strongly agree	46 (21.1)
Disagree/ strongly disagree	172 (78.9)
**I should go for a blood test once suspected of malaria**	
Agree/strongly agree	217 (99.5)
Neither agree nor disagree	1 (0.50)
**Sleeping Practices**	
**Did you sleep inside a LLIN net?**	
No	181 (83.0)
Yes	37 (17.0)
**Where do the women in your household sleep during menstruation? (n = 215)**	
Separate room in the house	211 (98.1)
Shed/ outside	4 (1.9)
**Do women in your household sleep under a LLIN net during menstruation? (n = 215)**	
No	138 (64.2)
Yes	77 (35.8)
